# Method for detection of hydrogen peroxide in HT22 cells

**DOI:** 10.1038/srep45673

**Published:** 2017-03-30

**Authors:** Dagmara Jacewicz, Kamila Siedlecka-Kroplewska, Joanna Drzeżdżon, Agnieszka Piotrowska, Dariusz Wyrzykowski, Aleksandra Tesmar, Krzysztof Żamojć, Lech Chmurzyński

**Affiliations:** 1 Faculty of Chemistry, University of Gdansk, Wita Stwosza 63, 80-308 Gdansk, Poland; 2 Department of Histology, Medical University of Gdansk, Debinki 1, 80-211, Gdansk, Poland

## Abstract

We have proposed a new method which can be applied in assessing the intracellular production of hydrogen peroxide. Using this assay we have examined the hydrogen peroxide generation during the L-glutamate induced oxidative stress in the HT22 hippocampal cells. The detection of hydrogen peroxide is based on two crucial reagents *cis*-[Cr(C_2_O_4_)(pm)(OH_2_)_2_]^+^ (pm denotes pyridoxamine) and 2-ketobutyrate. The results obtained indicate that the presented method can be a promising tool to detect hydrogen peroxide in biological samples, particularly in cellular experimental models.

Reactive oxygen species (ROS) are generated in cells under normal conditions e.g. during aerobic respiration^1^. Electrons transferred in the mitochondrial electron transport chain may escape and interact with molecular oxygen to form superoxide anion (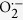)^1^. Superoxide dysmutase (SOD) catalyzes dismutation of 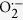 resulting in the generation of hydrogen peroxide (H_2_O_2_), which in turn can react with Fe^2+^ in the Fenton reaction to form hydroxyl radicals (HO^·^)^2^,^3^,^4^. When the cellular antioxidant defense mechanisms fail to effectively eliminate ROS the oxidative stress appears. Superoxide anion, hydroxyl radical or hydrogen peroxide lead to irreversible damage of lipids, proteins and DNA, resulting in dysfunction of cell organelles^5^,^6^. The extraordinary high rate of respiration in some organs such as the brain makes them particularly susceptible to ROS mediated injury. ROS and induced by them oxidative damage are believed to contribute to the development of many diseases such as cancer, arteriosclerosis, diabetes and neurodegenerative diseases^7^,^8^,^9^,^10^. Establishing the role of oxidative stress requires the ability to measure its mediators accurately^11^. Therefore, there is a need to develop and improve sensitive and specific methods to detect and evaluate the level of reactive oxygen species in biological samples of different origin. A method of protecting the neuronal cells HT22 against the oxidative stress generated by hydrogen peroxide has been investigated and described by Jia and co-workers^12^. This method is based on cytoprotection properties of endogenous cannabinoid anandamide. Moreover, recently it has been found that the Homer1a - protein regulating calcium pathways are involved in the glutamate-induced HT22 cell death^13^. Homer1a plays the cytoprotective role in the prevention of the glutamate-induced oxidative injury. In most cases existing methods to detect hydrogen peroxide in biological samples are based on the horseradish peroxidase (HRP) mediated reaction^14^. These methods are sensitive but have also disadvantages because HRP can react with many cellular substrates. Fluorescent probes used for the detection of H_2_O_2_ under biological conditions in addition to the advantages have some limitations. For example some probes require long response times (30–60 min) or an increase in the luminescence is also observed in the presence of the citrate and phosphate buffer^15^. Moreover, the autofluorescence of cells is a problem in the flow cytometry technique or fluorescence microscopy for the detection of H_2_O_2_. The purpose of this study was to design a method for the hydrogen peroxide detection which is characterized by a high sensitivity, moreover the autofluorescence of samples is eliminated and the results obtained by this method can be presented in concentration units.

The new method we propose is based on the following steps: (a) the nonenzymatic reaction of 2-ketobutyrate with H_2_O_2_ resulting in the CO_2_ release and (b) the interaction of the coordination compound cis-[Cr(C_2_O_4_)(pm)(OH_2_)_2_]^+^ with CO_2_. 2-ketobutyrate reacts with H_2_O_2_ with 1:1 stoichiometry (1). The amount of H_2_O_2_ in this reaction is equivalent with the CO_2_ released. 2-ketoacids such as 2-ketobutyrate or pyruvate can react nonenzymatically with H_2_O_2_, yielding CO_2_, H_2_O and the corresponding one carbon atom shorter carboxylic acid. This reaction was first reported by Holleman^16^.


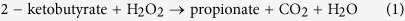


Thus, based on the CO_2_ measurement it is possible to assess the level of H_2_O_2_. The coordination complex of chromium [Cr(C_2_O_4_)(pm)(OH_2_)_2_]^+^, where pm denotes pyridoxamine, was shown to effectively catch CO_2_^17^. Previously we successfully used cis-[Cr(C_2_O_4_)(pm)(OH_2_)_2_]^+^ as a molecular biosensor to detect CO_2_ in osteosarcoma 143B cells treated with hydrogen peroxide^18^. In this study we demonstrate an improved version of this method based on both *cis*-[Cr(C_2_O_4_)(pm)(OH_2_)_2_]^+^ and 2-ketobutyrate.

## Results

To study the generation of hydrogen peroxide in a cellular model we used hippocampal HT22 cells treated with 5 mM L-glutamate for 24 h. Glutamate belongs to neurotransmitters of the central nervous system. However, at high (mM) concentration it was found to induce death of neurons^19^. 5 mM glutamate was reported to induce oxidative stress in HT22 cells resulting in cell death^20^. Our results also showed that the viability of HT22 cells treated with 5 mM L-glutamate for 24 h dramatically decreases (publication in press). On the basis of our proposed hydrogen peroxide detection method, we measured CO_2_ concentration after adding of 2-ketobutyrate. 2-ketobutyrate reacts with H_2_O_2_. The amount of CO_2_ released in this reaction reflects the amount of H_2_O_2_. In addition to cell lysates, the CO_2_ level was assessed in supernatants as well as in cell free culture medium – all of them potentially contained H_2_O_2_. It is important to note that they differed in chemical composition. In order to determine the most effective concentration of 2-ketobutyrate required to scavenge H_2_O_2_ we tested its following concentrations: 0.5 mM, 1 mM, 2 mM, 3 mM, 4 mM, 5 mM, 6 mM, 7 mM, 8 mM, 9 mM, 10 mM, 11 mM, 12 mM, 13 mM, 14 mM and 15 mM.

**Figure 1:**
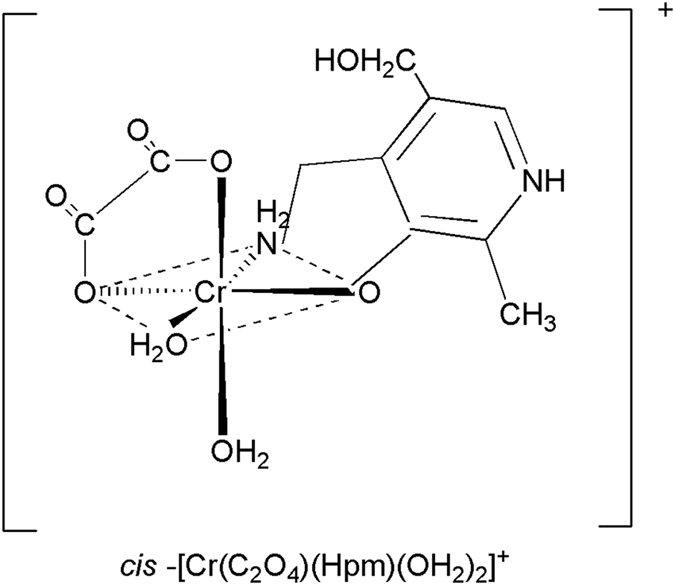
Structure of *cis*-[Cr(C_2_O_4_)(pm)(OH_2_)_2_]^+^.

In order to assess the CO_2_ concentration in samples we used a specific molecular biosensor *cis*-[Cr(C_2_O_4_)(pm)(OH_2_)_2_]^+^ and the spectrophotometric stopped-flow technique. The structure of the synthesized complex of chromium(III), *cis*-[Cr(C_2_O_4_)(pm)(OH_2_)_2_]^+^ ion, is shown in Fig. 1^21^.

It turned out that the coordination ion, *cis*-[Cr(C_2_O_4_)(pm)(OH_2_)_2_]^+^, could be successfully applied in the case of the detection of carbon dioxide generated in the reaction of decarboxylation of 2-ketobutyrate caused by H_2_O_2_. This reaction between the Cr(III) complex ion with pyridoxamine and the carbon dioxide was observed between 340–700 nm by using spectrophotometric stopped-flow method. The concentration of carbon dioxide has been identified based on the method which was used and described in the paper^22^.

**Figure 2:**
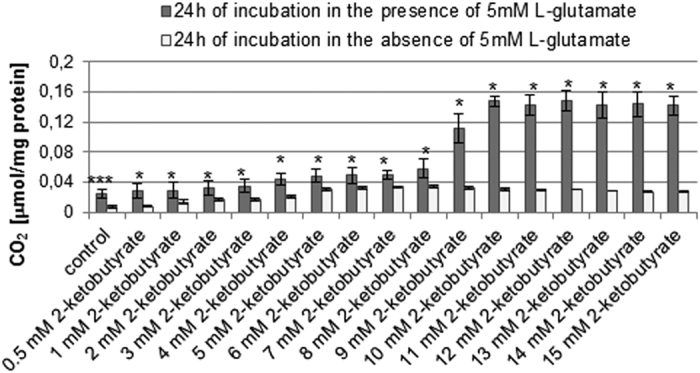
CO_2_ assessment in cell lysates. HT22 cells were incubated for 24 h in the presence or absence of 5 mM sodium L-glutamate. After treatment, cells were lysed. Before the CO_2_ measurement sodium 2-ketobutyrate was added (final concentations: 0.5–15 mM, respectively). The CO_2_ level was determined using *cis*-[Cr(C_2_O_4_)(pm)(OH_2_)_2_]^+^ and a stopped-flow technique. Data are presented as mean ± SD of three independent experiments. *p < 0.05, ***p < 0.001, statistically significant differences between samples incubated in the presence and samples incubated in the absence of 5 mM L-glutamate.

As shown in Fig. 2 the CO_2_ levels in lysates of cells incubated in the presence or absence of 5 mM L-glutamate, both measured without the addition of 2-ketobutyrate, were 0.025 μmol mg^−1^ protein (±0.006) and 0.006 μmol mg^−1^ protein (±0.002), respectively. We found that after addition of 0.5–10 mM 2-ketobutyrate to the lysates of L-glutamate-treated HT22 cells, the CO_2_ level gradually increased (Fig. 2). Noteworthy, at 2-ketobutyrate concentrations ranging from 10 mM to 15 mM the CO_2_ level was very similar (ranging from 0.14 μmol mg^−1^ protein to 0.15 μmol mg^−1^ protein). Moreover, the highest CO_2_ level was then observed. CO_2_ was also detected in lysates of cells incubated in the absence of 5 mM L-glutamate. However, its level was much lower than that in the presence of L-glutamate.

**Figure 3:**
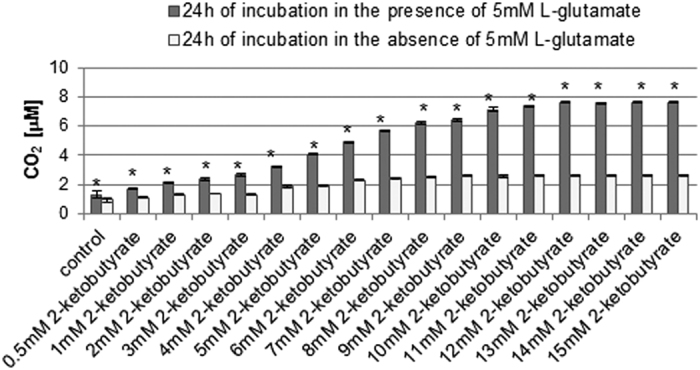
CO_2_ assessment in supernatants. HT22 cells were incubated for 24 h in the presence or absence of 5 mM sodium L-glutamate. After treatment, supernatants were separated from cells. Before the CO_2_ measurement sodium 2-ketobutyrate was added (final concentations: 0,5–15 mM, respectively). The CO_2_ level was determined using *cis*-[Cr(C_2_O_4_)(pm)(OH_2_)_2_]^+^ and a stopped-flow technique. Data are presented as mean ± SD of three independent experiments. *p < 0.05, statistically significant differences between samples incubated in the presence and samples incubated in the absence of 5 mM L-glutamate.

Intracellularly produced H_2_O_2_ has the ability to penetrate biological membranes and affect neighbouring cells^23^. In order to better evaluate and understand the chemical environment of cells in our experimental model, we assessed the CO_2_ level in supernatants. The results revealed that the CO_2_ levels in supernatants separated from cells incubated in the presence or absence of 5 mM L-glutamate, both measured without the addition of 2-ketobutyrate, were 1.315 μM (±0.220) and 0.949 μM (±0.114), respectively (Fig. 3). We found that the CO_2_ level gradually increased depending on the concentration of 2-ketobutyrate added (Fig. 3). In the case of supernatants separated from cells incubated in the presence of L-glutamate, at 2-ketobutyrate concentrations ranging from 10 mM to 15 mM the CO_2_ level was very similar and the highest (ranging from 7.15 μM to 7.6 μM). Moreover, the concentration of CO_2_ detected in supernatants separated from cells incubated in the absence of L-glutamate was then much lower than that in the presence of L-glutamate.

**Figure 4:**
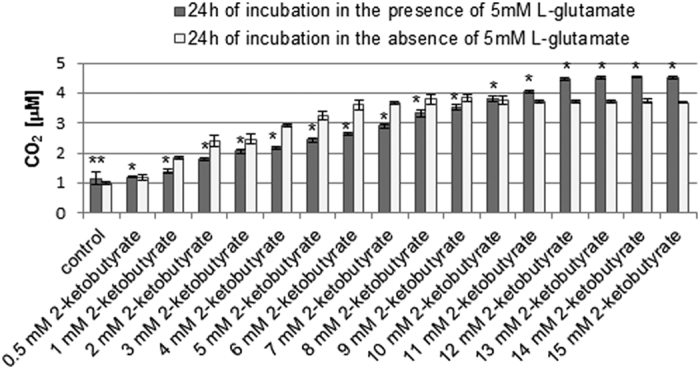
CO_2_ assessment in the cell-free complete culture medium. The cell-free culture medium was incubated for 24 h in the presence or absence of 5 mM sodium L-glutamate. After treatment, sodium 2-ketobutyrate was added (final concentations: 0.5–15 mM, respectively). The CO_2_ level was determined using *cis*-[Cr(C_2_O_4_)(pm)(OH_2_)_2_]^+^ and a stopped-flow technique. Data are presented as mean ± SD of three independent experiments. *p < 0.05, **p < 0.01 statistically significant differences between samples incubated in the presence and samples incubated in the absence of 5 mM L-glutamate.

The oxidation of chemical components of the culture medium may be a source of hydrogen peroxide generation^24^,^25^. The source of hydrogen peroxide in the cell-free DMEM medium, used in this study, in the absence of L-glutamate could be the oxidation of some chemical components of the culture medium. It has been reported that thiol compounds such as cysteine are unstable in culture media and can oxidize to generate hydrogen peroxide^24^. Noteworthy, cysteine is a constituent of the DMEM medium. There is also evidence that riboflavin may be a source of the photogeneration of ROS in cell culture media^25^. It has been shown that it is possible to generate superoxide anion and hydrogen peroxide as a product of its dismutation. Taking it into consideration, we have examined the CO_2_ level in the cell-free complete culture medium. The CO_2_ concentration in medium samples incubated in the presence or absence of 5 mM L-glutamate, both measured without the addition of 2-ketobutyrate, were 1.153 μM (±0.206) and 0.998 μM (±0.068), respectively (Fig. 4). We found that upon addition of 2-ketobutyrate at increasing concentrations, the CO_2_ level gradually increased (Fig. 4). The increase in CO_2_ concentration was observed in medium samples incubated in the presence or absence of 5 mM L-glutamate. Noteworthy, in the presence of L-glutamate after adding of 2-ketobutyrate at concentrations ranging from 12 mM to 15 mM the CO_2_ level was the highest and nearly constant (about 4.5 μM). In the absence of L-glutamate, at 2-ketobutyrate concentrations ranging from 6 mM to 15 mM only slight changes in the CO_2_ concentration appeared (ranging from 3.6 μM to 3.8 μM).

## Discussion

In this study, we demonstrate a new method of hydrogen peroxide detection. It can be applied to assess hydrogen peroxide level in different biological samples, e.g. cell lysates, supernatants or cell-free culture medium.

Our results suggest that the 10 mM–15 mM concentration range of 2-ketobutyrate can be used in the proposed method to effectively detect and assess the CO_2_ concentration in lysates of 5 mM L-glutamate-treated HT22 cells. At this concentration range the CO_2_ level was nearly the same. The main source of CO_2_ in cell lysates was then the reaction of 2-ketobutyrate with intracellularly produced H_2_O_2_. In order to find out whether the cell metabolism may affect the CO_2_ level, we incubated HT22 cells in the absence of 5 mM L-glutamate. We assume that in our experimental model CO_2_, whose main source are metabolic pathways, can be detected in the absence of L-glutamate (in the absence of H_2_O_2_ generating agents) and simultaneously in the absence of 2-ketobutyrate. Under these conditions CO_2_ does not originate from the reaction of 2-ketobytyrate with H_2_O_2_ as well as is not affected by L-glutamate, hence its main source can probably be metabolic pathways. Our results have shown that the CO_2_ level in lysates of cells incubated in the absence of 5 mM L-glutamate, measured without the addition of 2-ketobutyrate, is 0.006 μmol mg^−1^ protein (±0.002). It reflects the background CO_2_ level for lysates of cells nontreated with L-glutamate. The CO_2_ level measured in the absence of L-glutamate, but after the addition of 2-ketobutyrate includes CO_2_ originating from metabolic pathways. Another source can be endogenous H_2_O_2_ related to the function of some cell organelles, e.g. peroxisomes, mitochondria. Noteworthy, the CO_2_ level in lysates of cells incubated in the presence of 5 mM L-glutamate, measured without the addition of 2-ketobutyrate, is 0.025 μmol mg^−1^ protein (±0.006). It may be considered as the background CO_2_ level for lysates of cells treated with L-glutamate. It appears slightly higher than in the absence of 5 mM L-glutamate suggesting that 5 mM L-glutamate may affect the CO_2_ level. The background signal may be a disadvantage of the proposed method. However, a similar problem or disadvantage related to the background signal exists in many other experimental methods. Cellular autofluorescence, for example, resulting from natural fluorescence properties of some structural components of cells, affects the sensitivity of flow cytometry assays^26^,^27^,^28^. Autofluorescence of cells or tissues is also a problem in another widely used technique such as fluorescence microscopy^29^,^30^. The background fluorescence interferes with detection of specific fluorescent signals. In our proposed method the problem related to the background CO_2_ level in cell lysates can be solved. The possible solution could be to subtract the background signal from the analysis.

The results indicated that, the 10 mM–15 mM concentration range of 2-ketobutyrate is sufficient to assess the CO_2_ level in supernatants separated from 5 mM L-glutamate-treated HT22 cells. The CO_2_ level in the samples measured was then similar and stopped increasing. As mentioned previously, the main source of CO_2_ in supernatants was H_2_O_2_ released from the cells where it was produced^23^. However, the additional source of CO_2_ can also be H_2_O_2_ generated during oxidation of chemical components of the culture medium^24^,^25^. Therefore, we also determined the CO_2_ level in supernatants separated from cells incubated in the absence of 5 mM L-glutamate. We noticed that it was much lower than in the presence of 5 mM L-glutamate. CO_2_ detected in supernatants separated from cells nontreated with 5 mM L-glutamate, measured without the addition of 2-ketobutyrate, is 0.949 μM (±0.114) and corresponds to the background CO_2_ level in supernatants separated from cells nontreated with 5 mM L-glutamate. It results mostly from the oxidation of chemical components of the culture medium. CO_2_ detected in the supernatant separated from cells treated with 5 mM L-glutamate, measured without the addition of 2-ketobutyrate, is 1.315 μM (±0.220) and reflects the background CO_2_ level in supernatants separated from cells treated with 5 mM L-glutamate. Its source is related not only to the oxidation of chemical components of the culture medium, but also to the effects of L-glutamate on culture medium components.

We found that 2-ketobutyrate at a concentration from 12 mM to 15 mM can be used to assess the CO_2_ level in the cell-free culture medium incubated with 5 mM L-glutamate. The CO_2_ concentration was then about 0.8 μM higher in the presence of L-glutamate than in its absence, suggesting that 5 mM L-glutamate may generate a small amount of H_2_O_2_ and/or CO_2_ in the culture medium. The CO_2_ level (corresponding to H_2_O_2_) in the presence of L-glutamate reflects the oxidation of components of the culture medium as well as the influence of L-glutamate on culture medium components. The CO_2_ level measured without the addition of 2-ketobutyrate can be considered as the background CO_2_ level in the cell-free culture medium. In the presence of 5 mM L-glutamate it is 1.153 μM (±0.206), whereas in the absence of 5 mM L-glutamate 0.998 μM (±0.068). The background CO_2_ level may result mainly from the generation of CO_2_ during the oxidation/decomposition of some its components. The CO_2_ level in the presence of L-glutamate as well as the CO_2_ level in the absence of L-glutamate, both measured after addition of 2-ketobutyrate, include the background CO_2_ level. However, it is important to note that the CO_2_ level measured in cell lysates should not be affected by the background CO_2_ in culture medium/supernatants. Before analysis, cells are separated from the culture medium, washed and then lysed to obtain cell lysates.

It is important to note that cell lysates, supernatants and cell-free culture medium differ in chemical composition. Thus, they should be considered examples of different experimental models. The chemical composition of culture media is considered to resemble (at least in part) the natural cellular environment. It should imitate physiological conditions. However, further studies are needed to find out whether the method presented here can be applied to detect hydrogen peroxide in biological fluids, e.g. in the blood serum. Our results revealed that the concentration range of 2-ketobutyrate needed to assess the CO_2_ level in different models may not be the same. This highlights the importance of preliminary studies required to establish experimental conditions in every case. However, it is not a big disadvantage, because in many experiments it is a common procedure, e.g. a standard curve in protein measurements.

## Conclusions

The new method we propose can be useful in comparative studies, e.g. unknown samples can be compared to control samples. An advantage of this method is its high sensitivity. The lower limit of detection is equal to 10^−7^ M^22^. Another advantage of the method is that the results can be presented in concentration units. Majority of methods allow to use arbitrary instead of more precise concentration units, e.g. flow cytometry assays using 2′,7′- dichlorofluorescin diacetate or hydroethidine to measure intracellular production of reactive oxygen species^31^,^32^. In conclusion, the presented method based on both *cis*-[Cr(C_2_O_4_)(pm)(OH_2_)_2_]^+^ and 2-ketobutyrate seems to be a very promising tool to study the hydrogen peroxide generation.

## Methods

### Chemicals

Reagents: sodium 2-ketobutyrate and L-glutamic acid monosodium salt monohydrate were purchased from Sigma (USA). L-glutamic acid monosodium salt solutions were prepared in the sterile physiological saline solution before use. Each time sodium 2-ketobutyrate solutions before use were prepared in sterile water. C*is*-[Cr(C_2_O_4_)(pm)(OH_2_)_2_]^+^ ion was synthesized according to standard literature procedures. The final product - *cis*-[Cr(C_2_O_4_)(L-L)(O_2_CO)]^−^ (L-L denotes bidentate ligand – pyridoxamine (pm)) was prepared by the known method described in the literature^17^.

### Cell culture

The mouse hippocampal neuronal HT22 cell line was kindly provided by Professor T. Grune (Institute of Biological Chemistry and Nutrition, University Hohenheim, Stuttgart). HT22 cells were maintained in CO_2_ incubator, at 37** **°C in a humidified atmosphere containing 5% CO_2_. Cells were cultured in Dulbecco’s Modified Eagle’s Medium without sodium pyruvate (DMEM, Sigma-Aldrich, USA), supplemented with 10% heat-inactivated fetal bovine serum (Sigma-Aldrich, USA), 100 IU mL^-1^ penicillin (Sigma-Aldrich, USA) and 100 μg/mL streptomycin (Sigma-Aldrich, USA).

### Cell treatment and CO_2_ measurement

HT22 cells were seeded onto 6-well plates (2 × 10^5^ cells per well) and allowed to attach for 24 h. Cells were then incubated for 24 h in the presence or absence of 5 mM sodium L-glutamate. Additionally, the cell-free culture medium (DMEM) was incubated for 24 h with or without 5 mM sodium L-glutamate. After incubation, cells as well as cell culture medium in which the cells were incubated were collected and separated by centrifugation (300 × g, 5 min, room temperature). Next, HT22 cells were washed with PBS and suspended in a lysis buffer (0.15 M NaCl, 0.005 M EDTA, 1% Triton X-100, 0.01 M Tris-HCl). The cell-free complete culture medium was also collected. Sodium 2-ketobutyrate solution was then added (final concentration: 0.5 mM, 1 mM, 2 mM, 3 mM, 4 mM, 5 mM, 6 mM, 7 mM, 8 mM, 9 mM, 10 mM, 11 mM, 12 mM, 13 mM, 14 mM, 15 mM) to the samples (cell lysates, supernatants, cell-free culture medium), prior to CO_2_ measurement. The protein level in cell lysates was measured using Pierce BCA^TM^ Protein Assay Kit (Thermo Scientific, USA) according to the manufacturer’s instruction. The CO_2_ concentration was assessed using a spectrophotometric stopped-flow technique and a coordinate *cis*-[Cr(C_2_O_4_)(pm)(OH_2_)_2_]^+^ ion as a molecular biosensor of CO_2_.

### Statistical analysis

Statistical analysis was performed using the Statistica 9 software (StatSoft, Poland). Data are expressed as mean ± SD. Statistical differences were evaluated using the Mann-Whitney U test. Differences were considered significant at p < 0.05, p < 0.01, p < 0.001, respectively.

## Additional Information

**How to cite this article:** Jacewicz, D. *et al*. Method for detection of hydrogen peroxide in HT22 cells. *Sci. Rep.* **7**, 45673; doi: 10.1038/srep45673 (2017).

**Publisher's note:** Springer Nature remains neutral with regard to jurisdictional claims in published maps and institutional affiliations.
